# Canine invasive mammary carcinomas as models of human breast cancer. Part 1: natural history and prognostic factors

**DOI:** 10.1007/s10549-017-4548-2

**Published:** 2017-10-30

**Authors:** Frédérique Nguyen, Laura Peña, Catherine Ibisch, Delphine Loussouarn, Adelina Gama, Natascha Rieder, Anton Belousov, Mario Campone, Jérôme Abadie

**Affiliations:** 1 Oniris, Nantes Atlantic College of Veterinary Medicine Food Science and Engineering, Animal Cancers, Models for Research in Comparative Oncology (AMaROC) Research Unit, Site de La Chantrerie, 102 Route de Gachet, CS40706, 44307 Nantes, France; 2 CRCINA, INSERM, Université d’Angers, Université de Nantes, Nantes, France; 3 Department of Animal Medicine, Surgery and Pathology, Complutense University of Madrid, Madrid, Spain; 4 Department of Pathology, University Hospital, Nantes, France; 5 Animal and Veterinary Research Centre (CECAV), University of Trás-os-Montes and Alto Douro (UTAD), Vila Real, Portugal; 6 Pathology and Tissue Analytics, Pharma Research & Early Development, Roche Innovation Center Munich, Munich, Germany; 7 Pharmaceutical Sciences, Pharma Research & Early Development, Roche Innovation Center Munich, Munich, Germany; 8 Institut de Cancérologie de l’Ouest, Angers, France

**Keywords:** Dog, Spontaneous animal model, Breast cancer, Estrogen Receptor alpha, HER2, Prognosis

## Abstract

**Purpose:**

Dogs have been proposed as spontaneous animal models of human breast cancer, based on clinicopathologic similarities between canine and human mammary carcinomas. We hypothesized that a better knowledge of the natural history and prognostic factors of canine invasive mammary carcinomas would favor the design of preclinical trials using dogs as models of breast cancer.

**Methods:**

The 2-year outcome of 350 female dogs with spontaneous invasive mammary carcinoma was studied. The investigated prognostic factors included age at diagnosis, pathologic tumor size, pathologic nodal stage, lymphovascular invasion, histological grade, and expression of Estrogen Receptor alpha (ERα), Progesterone Receptor, Ki-67, Human Epidermal Growth Factor Receptor 2, basal cytokeratins 5/6, and Epidermal Growth Factor Receptor. Multivariate survival analyses were performed using the Cox proportional hazards model.

**Results:**

The overall survival after mastectomy was 11 months. Within 1 year post mastectomy, 41.5% of dogs (145/350) died from their mammary carcinoma. By multivariate analysis, the significant prognostic factors for overall survival included a pathologic tumor size larger than 20 mm [HR 1.47 (95% confidence interval 1.15–1.89)], a positive nodal stage [pN+, HR 1.89 (1.43–2.48)], a histological grade III [HR 1.32 (1.02–1.69)], ERα negativity [HR 1.39 (1.01–1.89)], a high Ki-67 proliferation index [HR 1.32 (1.04–1.67)], and EGFR absence [HR 1.33 (1.04–1.69)].

**Conclusion:**

The short natural history of spontaneous canine invasive mammary carcinomas and high rate of cancer-related death allow for rapid termination of preclinical investigations. The prognostic factors of invasive mammary carcinomas are remarkably similar in dogs and humans, highlighting the similarities in cancer biology between both species.

## Introduction

Breast cancer represents the most prevalent cancer and the leading cause of cancer death in women worldwide [1]. Despite considerable progress in breast cancer management, prognosis in the metastatic setting remains poor. The 5-year specific survival after initial diagnosis was estimated 97% for stage I, 88% for stage II, 70% for stage III, and only 25% for stage IV breast cancer [2]. One of the current challenges is to define molecular tools and relevant models that can predict the response and potential resistance to therapies. The classic in vitro (tumor cell lines) and in vivo (xenografts) preclinical models have indeed limitations related to the difficulty to reproduce interactions with the microenvironment, the absent or incomplete metastatic pattern, and their inability to fully integrate the host immune response [3]. Spontaneous tumor models are thus of high interest, to study the pharmacokinetics of innovative therapeutics in vivo, their effect on tumor (pathologic response) and patient (metastasis, survival), and the interactions between tumor cells and their microenvironment. In this respect, canine spontaneous cancers seem particularly relevant to human oncology [4–6].

Although their prevalence decreases in regions where early preventive ovariectomy is routinely performed, canine mammary carcinomas (CMCs) remain the most common canine cancer, with an estimated annual incidence of 182 per 100,000 female dogs [7]. Recent publications describe the relevance of spontaneous CMCs as models of human breast cancer, because of their high incidence, similarities in relative age of onset, risk factors, biological behavior, and metastatic pattern [8–11]. However, the biological behavior of CMCs needs further evaluation. Few studies dealt with the natural history of CMCs, i.e., the outcome of dogs after mastectomy as single therapy [12–17]. The prognostic factors of CMCs were poorly described, usually in medium-sized cohorts (45–229 dogs), and mostly by univariate analyses [15–29], although multivariate analyses are available [14, 30–41]. Because adjuvant chemotherapy does not significantly improve survival in dogs with advanced invasive CMC [41], and because tamoxifen-based hormone therapy is associated with significant adverse effects [42], most dogs benefit only from mastectomy, sometimes associated with ovariohysterectomy [43]. This situation allows studies of the natural history of invasive CMCs, and the identification of prognostic factors without the confounding effects of adjuvant therapy. This also favors preclinical therapeutic trials of new anti-cancer drugs as first-line regimens, rather than in relapsed patients with advanced cancer.

Here, we hypothesized that (1) knowledge of the natural history of CMCs would emphasize the aggressive and short course of the disease, and could be useful for the design of preclinical therapeutic trials in dogs with CMC, as translational models of human breast cancer; (2) knowledge of the prognostic factors of CMCs would highlight the biological similarities between spontaneous CMCs and breast cancers.

The aims of this study were thus to describe the natural history of invasive CMCs, i.e., cancer progression and mortality rates, in the largest cohort collected so far (350 female dogs); to describe invasive CMCs using human pathological criteria including immunohistochemical markers; and to validate these criteria as prognostic factors able to predict patients' outcome. In part 2 of this article, we evaluated the prognostic significance of the immunohistochemical classification of human breast cancer applied to dogs.

## Methods

### Patients and samples

This retrospective study included 350 female dogs with invasive mammary carcinoma, but free from other cancer, initially diagnosed in two laboratories of veterinary histopathology (Laboratoire d’Histopathologie Animale, Oniris, Nantes, and Laboratoire d’Anatomie Pathologique Vétérinaire d’Amboise, France) between 2007 and 2010. The owners’ written consent and approval from the Oniris College of Veterinary Medicine local Animal Welfare Committee were obtained prior to inclusion.

Dogs were eligible for inclusion when a histological diagnosis of invasive mammary carcinoma was established and confirmed by an absent layer of p63-positive myoepithelial cells (anti-p63 antibody, clone ab111449, Abcam) by immunohistochemistry (IHC) that differentiates invasive from in situ mammary carcinomas [44, 45]. All dogs were treated surgically by their veterinarian, and none of them received any additional treatment before and/or after mastectomy. Age, breed, spay status, parity, contraception, prior benign mammary lesions, medical history, and outcome were obtained through written questionnaires or telephone interviews with referring veterinarians and owners. All 350 dogs were followed for at least 48 months with particular emphasis on the occurrence of locoregional relapse (time between mastectomy and the earliest local recurrence on the same mammary gland, new primary mammary tumor, or lymph node metastasis), distant metastasis-free interval (time from mastectomy to first evidence of distant metastases by medical imaging), and disease-free interval (interval from mastectomy to the first local recurrence, new primary tumor, lymph node metastasis, and/or distant metastasis). Overall survival was defined as the time between mastectomy and death from any cause. Specific survival was defined as the time between mastectomy and death attributable to the mammary carcinoma.

### Pathological evaluation

Histological examination was performed on 3-μm-thick hematoxylin–eosin-saffron (HES) stained sections. The 350 tumors were classified according to the human breast cancer classification adapted to dogs (World Health Organization classification system) [46, 47], and graded according to the criteria of Elston and Ellis [48] adapted to canine mammary carcinomas [38]. The pathologic tumor size (pT, measured on histological slides), lymphovascular invasion (LVI), dermal infiltration, cutaneous ulceration, muscle invasion, margin status, and central necrosis were recorded for each case. Peritumoral lymphohistiocytic inflammation was considered positive when moderate to severe. In case of multicentric CMC, the largest tumor and/or tumor of highest histological grade was considered for prognostic purposes.

The methods used for IHC were detailed previously [35]. Briefly, automated IHC (Benchmark XT Ventana, Roche Diagnostics) was performed on 3-μm-thick serial sections using the following antibodies: monoclonal mouse anti-human Estrogen Receptor alpha (ERα, clone C311, Santa Cruz, dilution 1:50), monoclonal rabbit anti-human Progesterone Receptor (PR, clone 1E2, Roche Diagnostics, prediluted), monoclonal rabbit anti-Human Epidermal Growth Factor Receptor Type 2 (HER2, clone 4B5, Roche Diagnostics, prediluted), polyclonal rabbit anti-HER2 (Dako A0485, dilution 1:400), monoclonal mouse anti-human Ki-67 (clone MIB1, Dako, dilution 1:50), monoclonal mouse anti-human Cytokeratins 5/6 (CK5/6, clone D5/16B4, Dako, dilution 1:50), and monoclonal mouse anti-Epidermal Growth Factor Receptor Type 1 (EGFR, clone 31G7, Invitrogen, dilution 1:20).

ERα, PR, and Ki-67 were assessed based on the number of positive nuclei among > 500 neoplastic cells (manual image analysis, Image J software, National Institute of Health, Bethesda, Maryland, USA), and considered positive at threshold ≥ 10% for ERα and PR [45, 49], CK5/6, and EGFR [50]. The > 33.3% threshold for Ki-67 was evaluated by the receiver-operator-characteristic curve calculated for 2-year cancer-specific mortality. HER2 was scored 0 for no staining at all or incomplete, faint/barely perceptible membrane staining in ≤ 10% of tumor cells; 1 + for incomplete and faint/barely perceptible membrane staining in > 10% of tumor cells; 2 + for circumferential and incomplete and/or weak/moderate membrane staining in > 10% of tumor cells; or incomplete and circumferential membrane staining that is intense but within ≤ 10% of tumor cells; and 3 + for circumferential, complete, and intense membrane staining in > 10% of tumor cells. Carcinomas were considered HER2 positive only for a 3 + IHC score [45, 51].

Negative controls were included in each IHC run, and consisted in replacing the primary antibody with normal mouse or rabbit serum (prediluted reagents, Roche Diagnostics). The positive controls were mostly internal (epidermis and hair follicles for Ki-67, CK5/6, and EGFR; non-neoplastic mammary gland surrounding the carcinoma for ERα and PR; sebaceous glands for ERα). For HER2, the pathway HER2 4-in-1 control slides (Roche Diagnostics) were chosen to assess the quality of staining for each HER2 score (0, 1 + , 2 + , 3 +).

Four veterinary pathologists (JA, FN, LP, AG) and 1 medical pathologist (DL) examined the HES and IHC slides blindly (i.e., without any information on the dog or on the other pathologists’ interpretation). In case of discrepancy between evaluators, cases were collectively reviewed in order to achieve a consensual diagnosis, grade, and immunohistochemical scoring.

### Statistical analyses

The MedCalc^®^ statistical software (Ostend, Belgium) was used. Continuous variables are expressed as median, [range], mean ± standard deviation. Correlations between categorical variables were analyzed using the Pearson Chi-square test. The Kaplan–Meier method and log-rank tests were used for univariate survival analyses, and Cox proportional hazards models for multivariate survival analyses, whose results are reported using the Hazard Ratio (HR), its confidence interval (95%-CI), and the *p* value of each covariate. For all statistical tests, a *p* value < 0.05 was considered significant.

## Results

### Clinicopathologic features of invasive canine mammary carcinomas (CMCs)

The cohort comprises 350 female dogs with invasive CMC, including 253 (72.3%) intact and 97 (27.7%) spayed female dogs. The main characteristics of patients and CMCs are given in Table 1. The mean age at diagnosis was 11.0 ± 2.1 years [range (3.6–16.3), median 11.0 years]. Fifty-seven breeds were represented. Mixed-breed dogs (*n* = 78, 22.3%) outnumbered Poodles (*n* = 50; 14.3%), German Shepherds (*n* = 25; 7.1%), Brittany and Labrador Retrievers (*n* = 19 each; 5.4%), and Yorkshire Terriers (*n* = 10; 2.9%).

**Table 1:** Characteristics of dogs and their invasive mammary carcinoma at diagnosis

	*N*	%
Age (years) (*n* = 349)^a^		
< 11.7 years	229	65.6
≥ 11.7 years	120	34.4
Spay status (*n* = 350)		
Intact females	253	72.3
Neutered females	97	27.7
Multicentricity (*n* = 350)		
Single carcinoma	295	84.3
Multicentric carcinoma	55	15.7
Pathologic tumor size (*n* = 350)		
pT < 20 mm	141	40.3
pT ≥ 20 mm	209	59.7
Pathologic nodal stage (*n* = 350)		
pN0	39	11.1
pNX	236	67.4
pN+	75	21.4
Lymphovascular invasion (*n* = 350)		
LVI+	171	48.9
LVI–	179	51.1
Histological grade (*n* = 350)		
I	19	5.4
II	106	30.3
III	225	64.3
Histological type (*n* = 350)		
Invasive mammary carcinoma	350	100
Simple tubulopapillary	176	50.3
Simple solid	103	29.4
Complex	31	8.9
Anaplastic	21	6.0
Squamous cell	14	4.0
Inflammatory	5	1.4
Surgical margins (*n* = 350)		
Positive margins	158	45.1
Negative margins	192	54.9
Peritumoral inflammation (*n* = 350)		
Yes (moderate to severe)	168	48.0
No (absent to mild)	182	52.0
ERα (*n* = 350)		
ER+ (≥ 10%)	57	16.3
ER– (< 10%)	293	83.7
PR (*n* = 350)		
PR+ (≥ 10%)	40	11.4
PR– (< 10%)	310	88.6
Ki-67 (*n* = 350)		
Ki-67 low (≤ 33.3%)	162	46.3
Ki-67 high (> 33.3%)	188	53.7
HER2 clone 4B5 (*n* = 350)		
0	246	70.3
1 +	76	21.7
2 +	28	8.0
3 +	0	0
HER2 polyclonal Dako (*n* = 350)		
0	262	74.9
1 +	71	20.3
2 +	17	4.9
3 +	0	0
CK5/6 (*n* = 350)		
CK5/6+ (≥ 10%)	229	65.4
CK5/6– (< 10%)	121	34.6
EGFR (*n* = 350)		
EGFR+ (≥ 10%)	186	53.1
EGFR– (< 10%)	164	46.9

^a^One case with missing data

In 235 dogs (67.1%), the invasive carcinoma was the first mammary lesion detected, whereas 115 (32.9%) dogs had a history of previous non-malignant mammary lesions. Parity was unknown in 269 dogs (76.9%), and nulliparous females (*n* = 49; 14.0%) slightly outnumbered multiparous (*n* = 32; 9.1%) females. History of contraceptive use was reported in 20 (5.7%) dogs.

Tumors involved the abdominal and inguinal mammary glands (M3 to M5) in 256 cases (73.1%), the thoracic mammary glands (M1–M2) in 50 (14.3%), both in 11 (3.1%), and location was unrecorded in 33 cases (9.4%). The most common surgical procedure was radical mastectomy (excision of the 5 mammary glands of the affected side) in 156 dogs (44.6%), followed by regional (M1–M3 or M3–M5) mastectomy in 112 cases (32.0%), and single mastectomy in 64 cases (18.3%). Information on the surgical procedure was missing in 18 dogs (5.1%).

The mean pathologic tumor size was 18 ± 7 mm [median 18 mm, range (2–49), *n* = 227 dogs]; in the other cases, the pathologic tumor size could not be precisely determined due to larger size and/or positive margins. In 236 dogs (67.4%), the pathologic nodal stage was pNX due to absence of lymph node sampling for histopathology. Nodal stage pN+ (with metastasis of any size) was confirmed in 75 cases (21.4%). Six dogs (1.7%) had evidence of distant metastasis (M1) at diagnosis.

All of the included cases correspond to invasive mammary carcinomas according to breast cancer classification. The predominant histological types were simple tubulopapillary (*n* = 176; 50.3%), simple solid (*n* = 103; 29.4%), and complex carcinomas (malignant epithelial proliferation associated with benign myoepithelial proliferation, *n* = 31, 8.6%). The mean mitotic index was 41 ± 29 mitoses in 10 high-power fields [× 400, diameter of the field of view 0.55 mm; median 33, range (5–236)].

Regarding histopathological criteria of aggressiveness, dermal infiltration was present in 119 cases (34.0%), cutaneous ulceration in 50 cases (14.3%), abdominal or thoracic muscle infiltration in 65 cases (18.6%), peritumoral inflammation in 168 cases (48.0%), and central necrosis in 261 cases (74.6%).

**Fig. 1:**
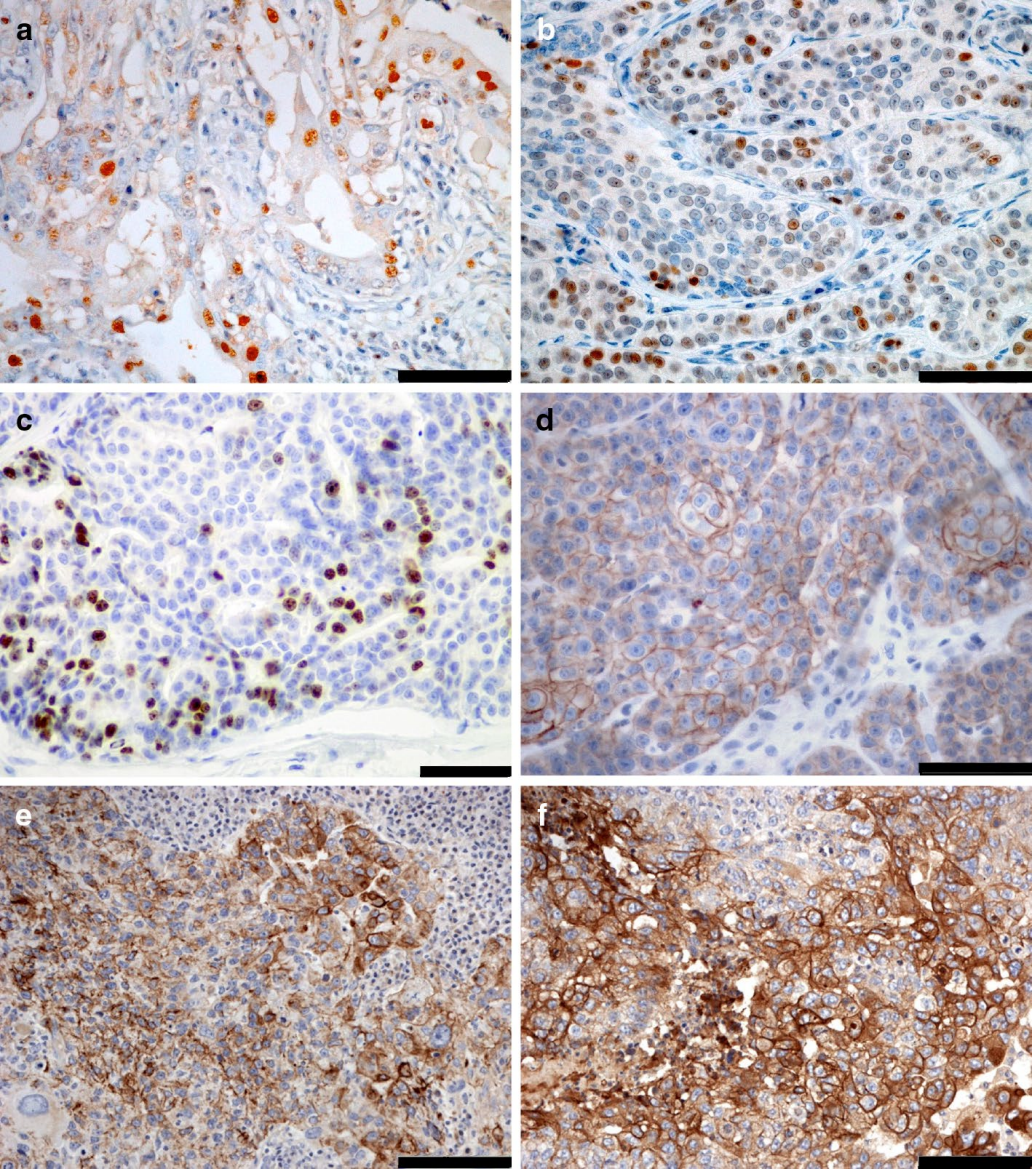
Immunohistochemical markers of canine invasive mammary carcinomas. Positivity to **a** Estrogen Receptor alpha (ERα, nuclear), **b** Progesterone Receptor (PR, nuclear), **c** the proliferation index Ki-67 (nuclear), **d** score 2 + for Human Epidermal Growth Factor Receptor type 2 (HER2, membranous), and positivity to **e** basal cytokeratins 5 and 6 (CK5/6, cytoplasmic), and **f** Epidermal Growth Factor Receptor type 1 (EGFR, membranous) in 6 different canine invasive mammary carcinomas. Indirect immunohistochemistry, initial magnification × 400, bar = 50 micrometers

The mean ERα index was 6.3 ± 14.0% (0–87.6%); 58.0% of cases (*n* = 203) did not express ERα at all. The mean PR index was 5.4 ± 14.8% (0–92.0%); 65.4% (*n* = 229) of CMCs did not express PR at all. At positive threshold 10%, 57 CMCs (16.3%) were ER + and 40 (11.4%) were PR+ (Fig. 1). Two hundred and sixty-seven CMCs (76.3%) were ER – PR − , 26 (7.4%) were ER − PR + , 43 (12.3%) were ER + PR − , and only 14 (4.0%) were ER + PR + . The mean Ki-67 index was 36.2 ± 17.4% [median 35.4%, range (1.3–94.6%)].

Both immunohistochemical protocols used to assess HER2 expression were highly correlated (*p* < 0.0001, Chi-square test). HER2 score 0 was predominant (70.3% of the cases with clone 4B5, 74.9% with polyclonal A0485), followed by HER2 score 1 + (Table 1). The cohort does not comprise any case with HER2 overexpression (score 3 +).

### Natural history and prognostic factors of canine invasive mammary carcinoma

#### Locoregional relapse (LRR)

**Fig. 2:**
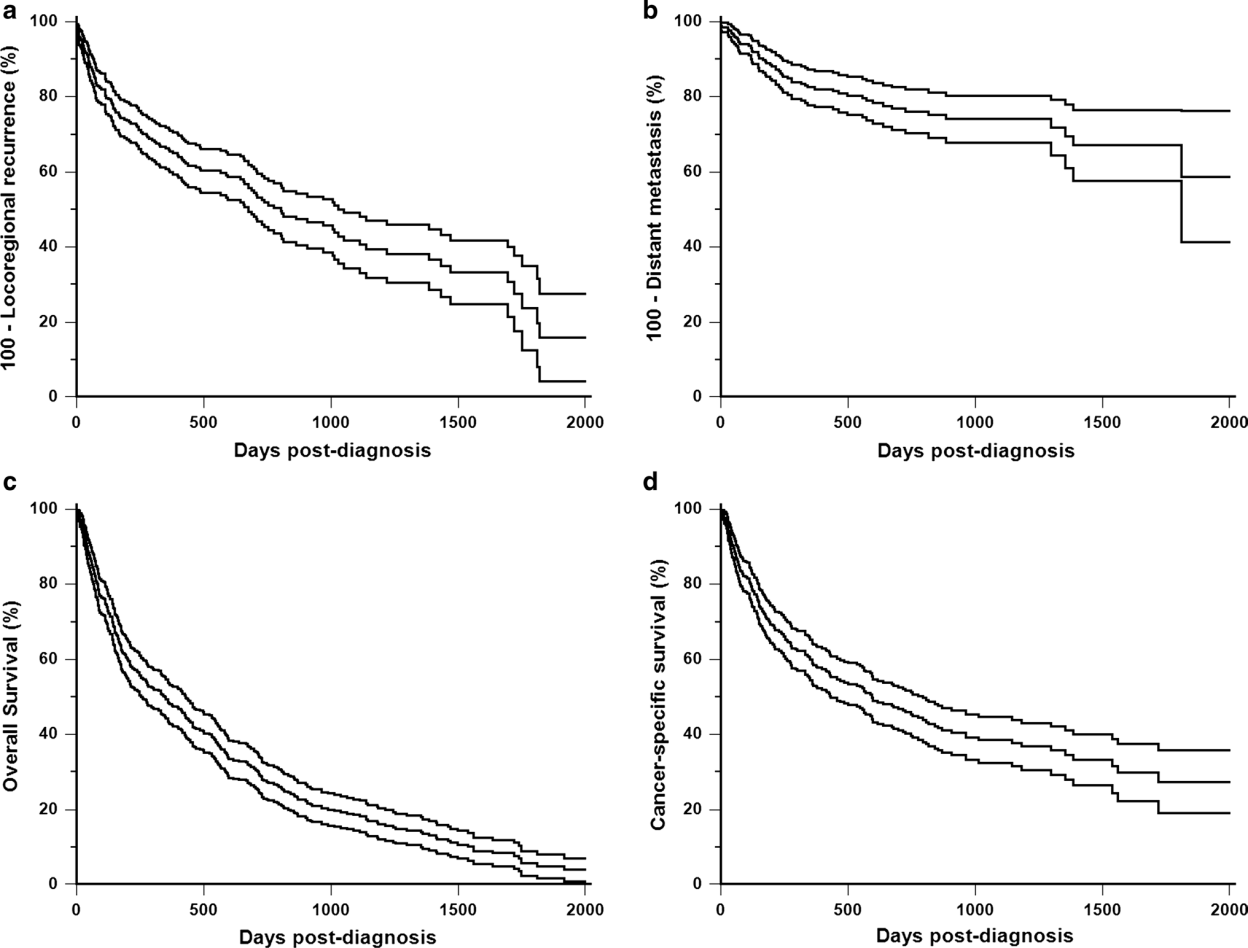
Natural history of invasive mammary carcinoma in 350 female dogs. Kaplan–Meier curves for **a** Locoregional Relapse (LRR), **b** Distant Metastasis-Free Interval (DMFI), **c** Overall Survival (OS), and **d** Specific Survival (SS). The 95% confidence interval is shown for each survival curve

The median time to LRR was 26.4 months; the LRR probability was 34% at 1 year, and 47% at 2 years post diagnosis (Fig. 2a). At the end of the follow-up period, 76 dogs (21.7%) had experienced tumor recurrence at the site of prior mastectomy, 56 dogs (16.0%) a new primary mammary tumor, and 18 dogs (5.1%) more than one locoregional event.

By univariate analysis, 11 parameters were significantly associated with the LRR risk (Table 2), of which 4 remained as significant independent prognostic factors by multivariate analysis (*p* < 0.0001): the strongest predictor of locoregional relapse was ERα positivity (HR 0.48), followed by the pathological nodal stage pN + (HR 1.92), the presence of lymphovascular invasion, and positive margins (HR = 1.55 for each).

**Table 2:** Prognostic factors for Locoregional Relapse of canine invasive mammary carcinomas by univariate and multivariate analyses

Univariate analysis	HR	95%-CI	*p*
Breed			
Molossoid breeds	2.26	1.10–4.64	0.0266
British and Irish pointing dogs	4.61	1.74–12.20	0.0022
Japanese, Chinese and Pekingese Spaniels	8.22	1.11–61.09	0.0406
Continental Toy Spaniels	11.56	1.52–87.73	0.0185
Molossian Toy dogs	16.40	2.17–123.95	0.0070
Any other breed	1.00	Reference	
Histological type			
Anaplastic CMC	2.38	1.19–4.77	0.0148
Inflammatory CMC	12.22	4.30–34.74	< 0.0001
Any other type	1.00	Reference	
Lymphovascular invasion			
LVI− versus LVI+	0.49	0.35–0.69	< 0.0001
Pathologic nodal stage			
pN+ versus pN0–pNX	2.31	1.46–3.67	< 0.0001
Margin status			
Positive versus negative margins	1.86	1.33–2.61	0.0001
Central necrosis			
Absent versus present	1.46	0.99–2.18	0.0343
Peritumoral inflammation			
No versus yes	0.67	0.48–0.92	0.0105
ERα			
ER+ versus ER−	0.49	0.33–0.73	0.0036
PR			
PR+ versus PR−	0.54	0.34–0.88	0.0460
Ki-67			
Continuous (%)	1.0107	1.0017–1.0197	0.0227
EGFR			
EGFR+ versus EGFR−	0.72	0.52–0.99	0.0428

Multivariate analysis			
Lymphovascular invasion			
LVI+ versus LVI−	1.55	1.08–2.24	0.0181
Pathologic nodal stage			
pN+ versus pN0–pNX	1.92	1.30–2.84	0.0012
Margin status			
Positive versus negative margins	1.55	1.10–2.18	0.0135
ERα			
ER+ versus ER−	0.48	0.29–0.79	0.0040

#### Distant Metastasis-Free Interval (DMFI)

The risk of distant metastasis was 17% at 1 year and 24% at 2 years post diagnosis (Fig. 2b), and was likely underestimated in this retrospective cohort, as the dogs’ owners may have declined complete staging, for financial reasons.

By univariate analysis, six parameters were significantly associated with DMFI (Table 3), of which four remained as significant independent prognostic factors by multivariate analysis (*p* < 0.0001): the strongest was lymphovascular invasion (HR 2.66), followed by age at diagnosis (HR 2.16 for older dogs), multicentricity (HR 1.89), and the Ki-67 proliferation index (HR 1.0149).

**Table 3:** Prognostic factors for Distant Metastasis-Free Interval (DMFI) of dogs with invasive mammary carcinomas (*n* = 350)

Univariate analysis	HR	95%-CI	*p*
Age at diagnosis			
≤ 11.7 versus > 11.7 years	0.44	0.25–0.75	0.0007
Multicentricity			
Single versus multicentric	0.44	0.20–0.96	0.0047
Lymphovascular invasion			
LVI− versus LVI+	0.33	0.20–0.56	<0.0001
Pathologic nodal stage			
pN+ versus pN0–pNX	1.86	0.92–3.75	0.0326
Peritumoral inflammation			
No versus yes	0.58	0.35–0.96	0.0281
Ki-67			
Continuous (%)	1.0203	1.0068–1.0339	0.0045

Multivariate analysis			
Age at diagnosis			
> 11.7 versus ≤ 11.7 years	2.16	1.29–3.62	0.0037
Multicentricity			
Multicentric versus single	1.89	1.03–3.46	0.0404
Lymphovascular invasion			
LVI+ versus LVI−	2.66	1.56–4.53	0.0003
Ki-67			
Continuous (%)	1.0149	1.0007–1.0293	0.0412

#### Disease-free interval (DFI)

The median DFI was 34.4 months. Cancer progression (locoregional recurrence and/or distant metastasis) was recorded in 34% of dogs at 1 year post diagnosis, and 45% at 2 years.

By univariate analysis, 13 parameters were significantly associated with DFI (Table 4), of which 4 were independent prognostic factors by multivariate analysis (*p* < 0.0001): the pathologic nodal stage pN + (HR 1.92), ERα negativity (HR 1.69), a high proliferation index (HR 1.59), and positive margins (HR 1.54).

**Table 4:** Prognostic factors for Disease-Free Interval of dogs with invasive mammary carcinomas (*n* = 350)

Univariate analysis	HR	95%-CI	*p*
Age at diagnosis			
Continuous (years)	1.1511	1.0624–1.2473	0.0006
Breed			
British and Irish pointing dogs	3.60	1.38–9.43	0.0094
Continental Toy Spaniels	22.64	5.10–100.46	< 0.0001
Molossian Toy dogs	15.41	2.04–116.71	0.0084
Any other breed	1.00	Reference	
Multicentricity			
Single versus multicentric	0.50	0.30–0.84	0.0007
Histological type			
Anaplastic CMC	2.11	1.01–4.38	0.0468
Inflammatory CMC	6.81	1.63–28.54	0.0090
Any other type	1.00	Reference	
Histological grade			
I versus II–III	0.36	0.15–0.89	0.0284
Lymphovascular invasion			
LVI− versus LVI+	0.35	0.25–0.49	< 0.0001
Pathologic nodal stage			
pN+ versus pN0–pNX	2.20	1.37–3.51	< 0.0001
Muscle invasion			
No versus yes	0.67	0.42–1.06	0.0482
Margin status			
Positive versus negative margins	1.63	1.15–2.30	0.0031
Peritumoral inflammation			
No versus yes	0.65	0.46–0.91	0.0093
ERα			
ER+ versus ER−	0.57	0.38–0.85	0.0222
Ki-67			
Continuous (%)	1.0164	1.0073–1.0256	0.0006
EGFR			
EGFR+ versus EGFR−	0.72	0.51–1.00	0.0473

Multivariate analysis			
Margin status			
Positive versus negative margins	1.54	1.09–2.16	0.0140
Pathologic nodal stage			
pN+ versus pN0–pNX	1.92	1.30–2.82	0.0011
ERα			
ER + versus ER−	0.59	0.36–0.97	0.0367
Ki-67			
≤ 33.3% versus > 33.3%	0.63	0.45–0.89	0.0096

#### Overall survival (OS)

During the follow-up period, 310 dogs (88.6%) died (Fig. 2c). The median OS was 11.4 months (2 days–75 months). The mortality rate was 51.7% at 1 year and 72.0% at 2 years post diagnosis. Death was unrelated to cancer in 58 dogs (16.6%), from unknown causes in 65 dogs (18.6%), and attributable to the invasive CMC in 187 dogs (53.4%).

By univariate analysis, 16 parameters were significantly associated with OS (Table 5), of which 6 were independent prognostic factors by multivariate analysis (*p* < 0.0001). The strongest prognostic factors were the pathologic nodal stage (pN + : HR 1.89) and pathologic tumor size (pT ≥ 20 mm: HR 1.47), followed by the histological grade, ERα positivity, the Ki-67 index, and EGFR expression (HR 1.32–1.39).

**Table 5:** Prognostic factors for Overall Survival of dogs with invasive mammary carcinomas (*n* = 350)

Univariate analysis	HR	95%-CI	*p*
Age at diagnosis			
Continuous (years)	1.1508	1.0898–1.2152	< 0.0001
History of contraception			
No versus yes/unknown	0.73	0.57–0.93	0.0106
Multicentricity			
Single versus multicentric	0.57	0.40–0.82	0.0001
Histological type			
Anaplastic CMC	3.45	2.18–5.46	< 0.0001
Inflammatory CMC	11.56	4.66–28.67	< 0.0001
Any other type	1.00	reference	
Histological grade			
I versus III	0.36	0.20–0.64	0.0006
II versus III	0.71	0.55–0.91	0.0066
Pathologic tumor size			
< 1 cm versus ≥ 2 cm	0.49	0.30–0.81	0.0054
1 cm ≤ pT < 2 cm versus ≥ 2 cm	0.65	0.51–0.83	0.0006
Lymphovascular invasion			
LVI– versus LVI+	0.42	0.34–0.54	< 0.0001
Pathologic nodal stage			
pN0 versus pNX	0.60	0.41–0.88	0.0091
pN+ versus pNX	1.80	1.38–2.36	< 0.0001
Dermal invasion			
No versus yes	0.77	0.60–0.98	0.0276
Muscle invasion			
No versus yes	0.66	0.48–0.91	0.0029
Margin status			
Positive versus negative margins	1.99	1.57–2.52	< 0.0001
Peritumoral inflammation			
No versus yes	0.68	0.54–0.85	0.0005
ERα			
ER+ versus ER−	0.69	0.53–0.91	0.0169
Ki-67			
≤ 33.3% versus > 33.3%	0.65	0.52–0.81	0.0001
CK5/6			
CK5/6+ versus CK5/6−	0.78	0.62–0.99	0.0376
EGFR			
EGFR > 0 versus EGFR absent	0.76	0.58–0.98	0.0217

Multivariate analysis			
Pathologic tumor size			
pT < 20 mm versus pT ≥ 20 mm	0.68	0.53–0.87	0.0026
Pathologic nodal stage			
pN+ versus pN0–pNX	1.89	1.43–2.48	< 0.0001
Histological grade			
I–II versus III	0.76	0.59–0.98	0.0328
ERα			
ER+ versus ER−	0.72	0.53–0.99	0.0436
Ki-67			
≤ 33.3% versus > 33.3%	0.76	0.60–0.96	0.0228
EGFR			
EGFR absent versus EGFR > 0	1.33	1.04–1.69	0.0239

#### Specific survival

The median time to death attributable to cancer was 19.5 months [2 days–56 months] (Fig. 2d). The cancer-related death rate was 41.5% at 1 year and 54.1% at 2 years post diagnosis.

By univariate analysis, 15 clinicopathologic parameters were significantly associated with cancer-related death (Table 6), of which six were independent prognostic factors by multivariate analysis (*p* < 0.0001). The most significant predictors of cancer-related death were those that define the stage of invasive CMCs: the pathologic tumor size (pT ≥ 20 mm: HR 1.41), pathologic nodal stage (pN + : HR = 1.82), and the presence of distant metastases at diagnosis (M1: HR 2.61). Peritumoral inflammation (HR 1.54), ERα negativity (HR 1.56), and a high Ki-67 proliferation index (HR 1.67) were also associated with cancer-related death, independently of the stage of the carcinoma at diagnosis.

**Table 6:** Prognostic factors for Cancer-Specific Survival of dogs with invasive mammary carcinomas (*n* = 350)

Univariate analysis	HR	95%-CI	*p*
Age at diagnosis			
Continuous (years)	1.1423	1.0652–1.2249	0.0002
Breed group			
Mixed-breed	1.98	1.09–3.62	0.0264
British and Irish pointing dogs	3.40	1.42–8.17	0.0065
Continental Toy Spaniels	24.39	5.62–105.81	< 0.0001
Any other breed	1.00	Reference	
Distant metastasis			
M1 versus M0–MX	3.19	0.77–13.18	0.0031
Multicentricity			
Single versus multicentric	0.50	0.32–0.78	0.0001
Histological type			
Anaplastic CMC	3.29	1.87–5.78	< 0.0001
Inflammatory CMC	14.35	5.71–36.07	< 0.0001
Any other type	1.00	Reference	
Histological grade			
I versus III	0.41	0.20–0.84	0.0151
II versus III	0.63	0.45–0.87	0.0054
Pathologic tumor size			
< 1 cm versus ≥ 2 cm	0.46	0.23–0.90	0.0251
1 cm ≤ pT < 2 cm versus ≥ 2 cm	0.70	0.51–0.95	0.0242
Lymphovascular invasion			
LVI− versus LVI+	0.31	0.23–0.42	< 0.0001
Pathologic nodal stage			
pN0 versus pNX	0.53	0.30–0.92	0.0242
pN + versus pNX	2.13	1.54–2.94	< 0.0001
Central necrosis			
No versus Yes	1.38	0.98–1.96	0.0444
Dermal invasion			
No versus Yes	0.74	0.54–1.00	0.0427
Margin status			
Positive versus negative margins	1.93	1.43–2.60	< 0.0001
Peritumoral inflammation			
No versus yes	0.59	0.44–0.79	0.0003
ERα			
ER + versus ER−	0.61	0.43–0.88	0.0209
Ki-67			
Continuous (%)	1.0179	1.0102–1.0257	< 0.0001

Multivariate analysis			
Pathologic tumor size			
pT < 20 mm versus pT ≥ 20 mm	0.71	0.52–0.95	0.0232
Pathologic nodal stage			
pN+ versus pN0–pNX	1.82	1.30–2.54	0.0005
Distant metastasis			
M1 versus M0–MX	2.61	1.14–5.99	0.0245
Peritumoral inflammation			
Yes versus no	1.54	1.14–2.07	0.0050
ERα			
ER+ versus ER−	0.64	0.41–0.97	0.0380
Ki–67			
≤ 33.3% versus > 33.3%	0.60	0.44–0.81	0.0011

## Discussion

Dogs with invasive mammary carcinomas have been proposed as a useful resource for preclinical research in comparative oncology due to epidemioclinical, biological, and pathological similarities with human breast cancer [8–11]. There was, however, a relative uncertainty of predictability of this spontaneous cancer as a translational model, as the natural history and prognostic factors have been described in relatively small cohorts [19–25, 27–41]. The present study is of particular interest because (1) mammary carcinomas in situ have been carefully excluded from analysis, using p63 immunohistochemistry when necessary, which is rarely, if ever, performed in veterinary studies, but of paramount importance in human breast oncology; (2) the cases were reviewed blindly by veterinary and medical pathologists, until consensus diagnoses were achieved, which permitted interpretation of canine samples using the criteria used for human breast cancer; (3) this is the largest cohort of CMCs described so far, which allowed for multivariate survival analyses with sufficient statistical power; (4) this study is one of the rare reports [14, 15, 17, 30, 37] of locoregional recurrence, distant metastasis-free interval, and specific survival in dogs with CMCs, as most previous studies focused on disease-free survival and overall survival only [18–21, 23–25, 28, 29, 31, 32, 35, 38, 39].

The epidemiological characteristics of CMCs in this cohort, although in agreement with some previous reports [20, 33], are characterized by an older age at diagnosis, lower rate of positivity to ERα and PR, and higher Ki-67 index than previous descriptions [19, 23, 27, 31, 37, 39]. These differences can be attributed at least in part to the systematic exclusion of mammary carcinomas in situ, which are diagnosed in younger dogs, and are more commonly ERα and PR positive compared to invasive CMCs (unpublished observations, manuscript in preparation), as described in human breast cancer [52].

Another particularity of this cohort is the absence of any HER2-positive CMC, as previously reported [53]. However, HER2-positive CMCs have been previously described by immunohistochemistry, with the polyclonal A0485 antibody [18, 26, 54–58] or the CB11 clone [59]. In this study, the external positive controls (cytospins of breast carcinoma cell lines, representative of the HER2 scores 0–3 +) ensured that HER2 expression was neither underestimated nor overestimated on the canine slides, a precaution that was rarely taken in veterinary oncology [26, 54]. Of note, HER2 gene amplification was not previously found in CMCs [60], and thus the existence of HER2-positive mammary carcinomas in dogs is still uncertain [61, 62].

The natural history of invasive CMCs is much shorter in dogs (54% cancer-related death at 2 years post diagnosis in this study) than in human breast cancer [2], probably in relation to shorter life expectancy in dogs, and the lack of adjuvant therapy, a situation that favors the setting of preclinical trials in the canine species. The effects of a given compound on patient survival, including in first-line regimen, is expected to be evaluable in short delays in dogs with CMCs, an advantage already highlighted for other canine cancers [4, 5]. The prognostic factors of invasive CMCs, described here in the largest retrospective cohort described so far, include the pathologic tumor size, pathologic nodal stage, lymphovascular invasion, histological grade, and ERα positivity, which are all also strong prognostic factors in human breast cancer [63], confirming the similar biology of invasive mammary carcinomas in both species.

## Conclusions

The results of the present study confirm that canine invasive mammary carcinomas have a short disease course, which is predictable with clinicopathologic criteria close to those of human oncology. In the second part of this article, we hypothesized that CMCs could be subdivided into luminal and triple-negative cases with different outcomes, as in human breast cancer.

## List of Abbreviations

95%-CI: 95% confidence interval
CK5/6: Cytokeratins 5 and 6
CMC: Canine mammary carcinoma
DFI: Disease-free interval
DMFI: Distant metastasis-free interval
EGFR: Epidermal growth factor receptor (type 1)
ERα: Estrogen receptor alpha
HER2: Human epidermal growth factor receptor type 2
HES: Hematoxylin–eosin-saffron
HR: Hazard ratio
IHC: Immunohistochemistry
LRR: Locoregional relapse
LVI: Lymphovascular invasion
M: Distant metastasis
OS: Overall survival
PR: Progesterone Receptor
pT: Pathologic tumor size
pN: Pathologic nodal stage
SS: Specific survival
